# Involvement of Histone Lysine Crotonylation in the Regulation of Nerve-Injury-Induced Neuropathic Pain

**DOI:** 10.3389/fimmu.2022.885685

**Published:** 2022-07-14

**Authors:** Yu Zou, Xue-Hui Bai, Ling-Chi Kong, Fei-Fei Xu, Ting-Yu Ding, Peng-Fei Zhang, Fu-Lu Dong, Yue-Juan Ling, Bao-Chun Jiang

**Affiliations:** ^1^ Pain Research Laboratory, Institute of Nautical Medicine, Nantong University, Nantong, China; ^2^ Medical School of Nantong University, Nantong, China; ^3^ Research Center of Clinical Medicine, Affiliated Hospital of Nantong University, Nantong, China

**Keywords:** histone crotonylation, neuropathic pain, partial infraorbital nerve transection, inflammatory cytokines, chemokine, macrophage

## Abstract

Histone lysine crotonylation (KCR), a novel epigenetic modification, is important in regulating a broad spectrum of biological processes and various diseases. However, whether KCR is involved in neuropathic pain remains to be elucidated. We found KCR occurs in macrophages, sensory neurons, and satellite glial cells of trigeminal ganglia (TG), neurons, astrocytes, and microglia of the medulla oblongata. KCR in TG was detected mainly in small and medium sensory neurons, to a lesser extent in large neurons. Peripheral nerve injury elevated KCR levels in macrophages in the trigeminal and dorsal root ganglia and microglia in the medulla oblongata but reduced KCR levels in sensory neurons. Inhibition of histone crotonyltransferases (p300) by intra-TG or intrathecal administration of C646 significantly alleviated partial infraorbital nerve transection (pIONT)- or spinal nerve ligation (SNL)-induced mechanical allodynia and thermal hyperalgesia. Intra-TG or intrathecal administration of Crotonyl coenzyme A trilithium salt to upregulate KCR dose-dependently induced mechanical allodynia and thermal hyperalgesia in mice. Mechanismly, inhibition of p300 alleviated pIONT-induced macrophage activation and reduced the expression of pain-related inflammatory cytokines *Tnfα*, *Il1β* and chemokines *Ccl2* and *Cxcl10*. Correspondingly, exogenous crotonyl-CoA induced macrophage activation and the expression of *Tnfα*, *Il1β*, *Il6*, *Ccl2* and *Ccl7* in TG, which C646 can repress. These findings suggest that *histone crotonylation* might be functionally involved in neuropathic pain and neuroinflammation regulation.

## Introduction

Nerve pressure- or nerve damage-induced neuropathic pain frequently occurs after surgery or trauma. This intractable pain will progress over months to years, even after curing the initial injury (1). Neuropathic pain is difficult to achieve relief. In some cases, opioid drugs are the only option for treatment. Despite promising beneficial effects, opioid drugs are associated with severe side effects, including respiratory depression, nausea, addiction, and analgesic tolerance (2). Therefore, further understanding the molecular mechanism of neuropathic pain is necessary and searching for new targets and therapies. Recent studies have shown that the altered gene expression profile in sensory ganglia after peripheral nerve injury is fundamental to the development of peripheral sensitization (3). Epigenetic regulation, such as DNA methylation and non-coding RNA, plays a vital role in regulating neuropathic pain *via* shaping the transcriptomes of DRG or spinal dorsal horn (SDH) in response to nerve injury (4–6). However, whether other forms of epigenetic regulation are involved in the regulation of neuropathic pain need to be explored.

Histone lysine crotonylation (KCR), a newly identified epigenetic modification, is a positive transcription regulator associated with active chromatin (7, 8). p300 has also been proved to catalyze histone crotonylation, which directly stimulates transcription even greater than histone acetylation (9). Conversely, Class I histone deacetylases (HDACs) rather than sirtuin family deacetylases were the major direct histone decrotonylases (10). Evolutionarily conserved YEATS domain as a family of crotonyllysine readers, directly linking histone crotonylation to active transcription (11). KCR level correlates tightly with the state of cell metabolism. There was a positive association between the cellular concentration of crotonyl-CoA and gene expression (9, 12). Chromodomain Y-like transcription corepressor CDYL plays an undirect suppressive role on KCR *via* acting as a crotonyl-CoA hydratase to convert crotonyl-CoA to β-hydroxybutyryl-CoA (13). Recent research has demonstrated the involvement of KCR in critical processes, such as acute kidney injury (AKI), Spermatogenesis, HIV Latency, DNA Damage Response and commitment of embryonic stem cells (13–17). In the nervous system, the level of KCR in the medial prefrontal cortex (mPFC) is associated with social defeat stress (CSDS) induced depression (18). However, whether KCR occurs in the peripheral nervous system and spinal cord and whether it participates in pain onset and progression is unclear.

Here, we performed preliminary research on the effect of histone lysine crotonylation on neuropathic pain. This study systematically examined KCR in normal and disease contexts in mice’s DRG, TG, and medulla oblongata tissues. We showed that the administration of crotonyl-CoA induced neuropathic pain and neuroinflammation. We also showed that inhibition KCR writer p300 attenuated partial infraorbital nerve transection (pIONT)- or spinal nerve ligation (SNL)-induced neuropathic pain. Overall, our results demonstrate the involvement of KCR in neuropathic pain for the first time.

## Materials and Methods

### Animals and Surgery

Experimental Animal Center of Nantong University provided adult male ICR mice weighing 22-28 g. The animals were maintained under controlled temperature (23 ± 1°C) and light (12-h light/dark cycle) with free access to food and water. *Cx3cr1^GFP^* mice (005582, B6.129P2(Cg)-*Cx3cr1^tm1Litt^*/J) were obtained from Jackson Lab (Bar Harbor, ME, USA). The pIONT, SNL and their respective sham mice models were produced followed previously described methods (19, 20). A mouse model of inflammatory pain was induced by subcutaneous intraplantar injection of a single dose of CFA (20 μl, Sigma, St Louis, MO), performed as described previously (21). All animal procedures were conducted using protocols following the National Institutes of Health guide for the care and use of laboratory animals (NIH Publications No. 8023, revised 1978). The animal study was also reviewed and approved by the Ethics Committee of Nantong University (S 20190802-004).

### Drugs and Administration

p300 inhibitor C646 and Crotonyl coenzyme A trilithium salt (Crotonyl-CoA Lithium) were purchased from Selleck Chemicals (S7152, Houston, USA) and Sigma-Aldrich (28007, USA), respectively. Minocycline hydrochloride was purchased from Merck Millipore (475843, Merck Millipore, USA). The intra-TG and intrathecal injection (IT) were performed as described previously under isoflurane anesthesia (19, 20). For intra-TG injection, 3 dose groups of Crotonyl-CoA Lithium (0.2 µg, 1 µg and 2 µg) and 3 dose groups of C646 (0.008 µg, 0.04 µg and 0.2 µg) were solved in 6 µl PBS and was applied to age- and body size-matched naive and pIONT mice, while PBS was used as control. Each mouse in each dose group received a single injection of 6 μL. For intra-TG injection of minocycline hydrochloride, 1 nmol minocycline was injected with 2 µg Crotonyl-CoA Lithium. The intrathecal injection was made with a 30 G needle between the L5 and L6 intervertebral spaces to deliver the reagents to the cerebral spinal fluid. Each animal received 1 injection of 10 μL regent (2 µg Crotonyl-CoA Lithium, or 0.2 µg C646). For intraplantar injection, 3 dose groups of Crotonyl-CoA Lithium (1 µg, 5 µg, and 10 µg in 20 µl PBS) were injected into the left hind paw of the animal. At the same time, the normal control mice had intraplantar injection with 20 µl PBS.

### Real-Time Quantitative PCR for mRNAs

For mRNA detection, the total RNAs of TG were extracted using Trizol reagent (Invitrogen, CA, USA). 1μg of total RNA was reverse transcribed using an oligo (dT) primer according to the manufacturer’s protocol of a HiScript^®^ III 1st Strand cDNA Synthesis Kit (Vazyme Biotech, Nanjing, China). qPCR analysis was performed in the Real-time Detection System (ABI StepOne Plus cycler, Foster City, USA) by TaKaRa TB Green Premix Ex Taq II (Takara, Dalian, China). The detailed primer sequences for *Tnfα*, *Il1β*, *Il6, Ccl2, Ccl 7, Cxcl 10, Iba-1 and Gapdh* are as follows: *Tnf-α* forward, 5’-GTT CTA TGG CCC AGA CCC TCA C-3’, and reverse, 5’-GGC ACC ACT AGT TGG TTG TCT TTG-3’; *Il1β* forward, 5’-TCC AGG ATG AGG ACA TGA GCA C-3’, and reverse, 5’- GAA CGT CAC ACA CCA GCA GGT TA -3’; *Il6* forward, 5’-TAG TGG ATG CTA CCA AAC TGG A-3’, reverse, 5’-TGT GAC TCC AGC TTA TCT CTT G G-3’; *Ccl2* forward, 5’-TGC TGC TAC TCA TTC ACT GGC-3’, and reverse, 5’-CCT TAT TGG GGT CAG CAC AG-3’; *Ccl 7* forward, 5’-TTT TGG TGG GTT TTG AAC AT-3’, and reverse, 5’-TGC TTC CAT AGG GAC ATC AT-3’; *Cxcl10* forward, 5’-TGA ATC CGG AAT CTA AGA CCA TCA A-3’; and reverse, 5’-AGG ACT AGC CAT CCA CTG GGT AAA G-3’; *Gapdh* forward, 5’-AAA TGG TGA AGG TCG GTG TGA AC-3’, and reverse, 5’-CAA CAA TCT CCA CTT TGC CAC TG-3’. The PCR amplifications were performed at 95°C for 30 s, followed by 40 cycles of thermal cycling at 95°C for 5 s and 60°C for 45 s. *Gapdh* was used as an endogenous control to normalize differences for mRNA. Quantification was performed by normalizing Ct (cycle threshold) values with *Gapdh* Ct (mRNA) and analyzed with the 2^-ΔΔCT^ method.

### Immunohistochemistry

Animals were anesthetized by isoflurane followed by perfusion *via* the ascending aorta with saline and 4% PFA. After perfusion, the associated tissue was removed and fixed in 4% PFA overnight at 4°C. Finally, the tissue was dehydrated in a 20% and 30% sucrose solution gradient. TG, DRG (15 μm), medulla oblongata and spinal cord sections (30 μm) were cut in a cryostat and processed for immunofluorescence as we described previously (19, 20). The antibodies information was listed as follows: KCR (rabbit, 1:1000, PTM BIO), GS (mouse, 1:500, Millipore), IBA-1 (goat, 1:500, Abcam), TUJ1 (mouse, 1:500, CST), NF200 (mouse, 1:500, Sigma), CGRP (goat, 1:3000, Millipore), IB4 (1:300, Sigma), NeuN (mouse, 1:1000, Millipore), GFAP (mouse, 1:5000, Millipore) and phosphorylated ERK (p-ERK, rabbit, 1:1000, CST). FITC- or Cy3-conjugated secondary antibody (1:400, Jackson ImmunoResearch, West Grove, PA). To detect the cellular localization of KCR, the sections were incubated with primary antibodies overnight at 4°C, then incubated by a mixture of FITC- and Cy3-conjugated secondary antibodies for 1 h at room temperature. After immunostaining, sections were counterstained with DAPI and examined with a confocal laser scanning microscopy (SP8, Leica, Germany) or a Nikon Electron Microscope (Nikon Eclipse NiE, Japan).

### Quantification of Immunofluorescence Staining

Fluorescence signals were quantified using Image J software. The detailed method was described as follows. The linear shape/selection tool was used to trace the scale length, choosing “Set Scale” from the analysis menu and entering the known distance and unit of length. Then, selecting the target cell was done using the drawing/selection tools of the polygon. Finally, measurements were set to Area, Integrated Density, and Mean grey value from the analyze menu. The number of KCR^+^/IBA-1^+^ cells was obtained by counting the IBA-1 and KCR co-labeled cells within an arbitrary unit area. To obtain the mean intensity of KCR in IBA-1^+^ cells, TUJ1^+^ cells and GS^+^ Cells, 3 mice per group were analyzed, and for each mouse, 3 sections per mouse and 30 cells per section were evaluated. To obtain the mean intensity of KCR among TUJ1^+^ cells, its intensity was quantified in a random field in each of three slices per mouse (3mouse per group).

### Behavioral tests

All behavioral experiments were conducted by experimenters unaware of the treatment and treatment groupings.

#### Trigeminal Neuropathic Pain Behavioral Test

Mice were habituated to the testing environment for at least two days before baseline testing. All the behavioral experimenters were done by individuals that were blinded to the different treatments of the mice. Animals were acclimated in metal mesh boxes for 30 min before the examination to test facial pain behavior. 0.02 g and 0.16 g von Frey filaments, respectively, were used to assess facial mechanical allodynia in the ipsilateral infraorbital nerve region, as we previously reported (19). The nociceptive behavior was scored as follows; 0, animal did not respond or looked around; 1, animal responded mildly or withdrew its face; 2, animal quickly retracted its face or raised its paw slightly; 3, animal displayed robust and agile avoidance behavior and raised the action of rubbing his face with his claws; 4, The animal exhibited the behavior of wiping and raising the paw, but the number of swabbing was less than 3 times; 5, The animal exhibited wiping and raising the paw and wiping more than 3 times.

#### SNL- and CFA- Induced Pain Test

To test the mechanical sensitivity of the hind paw ipsilateral plantar surface to the SNL, animals were put in boxes on an elevated metal mesh floor and allowed 30 min for habituation before the examination. The plantar surface of each hind paw was stimulated with a series of von Frey hairs with logarithmically incrementing stiffness (0.02-2.56 g, Stoelting, Wood Dale, IL), presented perpendicular to the plantar surface (2 to 3s for each hair). Dixon’s up-down method determined the 50% paw withdrawal threshold (22). For testing heat sensitivity, animals were put in plastic boxes and allowed 30 min for habituation. Heat sensitivity was tested by radiant heat using the Hargreaves apparatus (IITC Life Science Inc., Woodland Hills, CA) and expressed as paw withdrawal latency (PWL). The radiant heat intensity was adjusted to 30% of the maximal so that basal PWL is between 10 and 12s, with a cut-off of 20 s to prevent tissue damage (23).

#### Tail Immersion Test

The water temperature got set to 48, 50, or 52°C. The latency period of the tail dip was recorded. To avoid the potential for injury, the cut-off was set to 10 seconds (20).

#### Rota-Rod Test

Mice were trained at 10 rpm for 3 minutes on a spinning rod until the mice no longer fell off the rod. For the test, the speed was set at 10 rpm for 60 seconds and then accelerated to 80 rpm over 5 minutes. The time taken for the mice to fall after the start of acceleration was recorded (20).

#### Nociceptive Behavior Test

The test was carried out regarding the previous formalin test (24). Immediately after injection, the mice were individually placed back in the clear cage. A video camera was positioned under the cage for recording. Behaviors such as licking, biting, lifting, and flinching, were observed for 60 min at 5 min intervals using a stopwatch in individual cages.

### Statistical Analysis

All data were expressed as mean ± SEM. Differences between groups were compared using one-way ANOVA or two-way ANOVA followed by Bonferroni’s test or using *student*’s t-test (two-tailed) if only two groups were applied. The criterion for statistical significance was *P* < 0.05. GraphPad Prism 8.0 graph the data and calculate all the tests. BioRender online software created the illustration (https://biorender.com/).

## Results

### Expression and Cellular Localization of KCR in TG and Medulla Oblongata From Normal Mice

**Figure 1:**
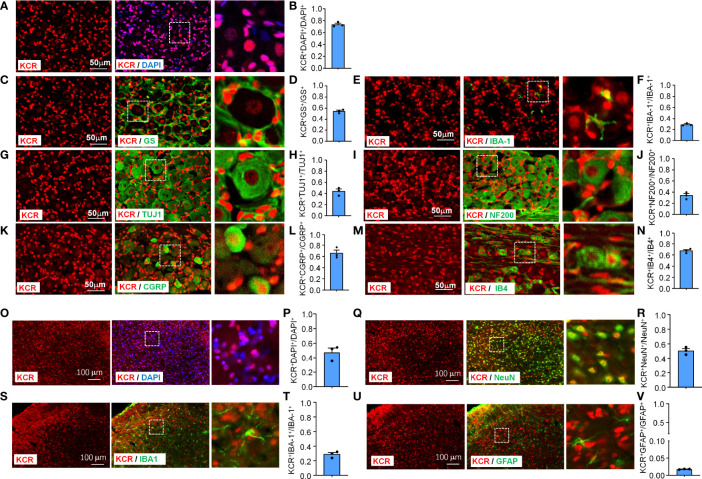
Immunofluorescence detection of KCR distribution and expression in TG (Scale bar, 50 μm) and medulla oblongata (Scale bar, 100 μm). The boxed region of the merged images was enlarged on the right. Bar graphs represent ratios plotted as mean ± SEM, n = 3 sections per mouse from 3 mice per group. **(A, B)** DAPI and KCR staining show the ratio of KCR-positive cells to all cells in TG. **(C–H)** Double staining immunofluorescent images show the distribution of KCR in satellite cells **(**GS, **C, D)**, macrophages **(**IBA–1, **E, F)** and sensory neurons **(**TUJ1, **G, H)** in TG of naive mice. The results showed that KCR occurs in neurons, satellite cells and microglia. **(I–N)** Immunofluorescent images show colocalization of KCR with NF200 **(**large myelinated neuron marker, **I, J)**, CGRP **(**small peptidergic neuron marker, **K, L)** and IB4 **(**small, nonpeptidergic neuron marker, **M, N)** in TG of naive mice. The results showed that KCR was mainly colocalized with small and medium neuron markers and a minority with large size neuron markers. **(O, P)** DAPI and KCR staining show the ratio of KCR-positive cells to all cells in the medulla oblongata. **(Q–V)** Representative photomicrographs show double fluorescence labeling directed to KCR (red)/NeuN **(**green, sensory neuron marker, **Q, R)**, KCR/IBA-1 **(**green, microglia marker, **S, T)** and KCR/GFAP **(**green, astrocyte marker, **U, V)** in the medulla oblongata, scale bar = 100 μm. The results showed that KCR was colocalized primarily with neurons and microglia and a minority with astrocytes.

To characterize the pattern of histone crotonylation in TG, we performed double-label immunofluorescence of KCR with DAPI, a pan-sensory neuron-specific marker TUJ1, a satellite glia cell marker GS, and a pan-macrophage marker IBA-1. DAPI staining was used to delineate the shape of nuclei from different cell types. We found that the KCR signals are diffuse throughout the nucleus (**Figure 1A**). Approximately 74% of TG cells showed positive KCR staining (**Figure 1A, B**). The fluorescence signal of KCR was detected in all three cell types in TG (**Figures 1C–H**). The fluorescence intensity of KCR in the majority of sensory neurons appeared weaker than that in macrophages or satellite cells (**Figures 1C–H**). To determine which subpopulation of sensory neurons are prone to histone crotonylation, the TG sections were permeabilized and double-labeled with KCR and NF200 (a marker of myelinated A-fibers), CGRP (a marker of peptidergic C-fibers) and IB4 (a marker for nonpeptidergic C-fibers), respectively. KCR is distributed in all neuron types (**Figures 1I–N**). The quantitative analysis revealed that KCR mainly occurred in small and medium sensory neurons labeled with CGRP and IB4 (**Figures 1I–N**).

The medulla oblongata plays an important role in trigeminal neuropathic pain processing and transmission (25). We thus examined the cellular distribution and expression of KCR in the medulla oblongata. KCR was detected in about 47% of medulla oblongata cells (**Figures 1O, P**). The immunofluorescence results revealed that KCR was colocalized predominantly with neuron marker NeuN (**Figures 1Q, R**), or microglia marker IBA-1 (**Figures 1S, T**), and to a lesser extent with astrocyte marker GFAP (**Figures 1U, V**) in the medulla oblongata. These results suggest that neurons and microglia are the major cell types responsible for KCR occurring in the medulla oblongata.

### Nerve Injury Altered the Levels of KCR in TG Macrophage and Sensory Neurons

**Figure 2:**
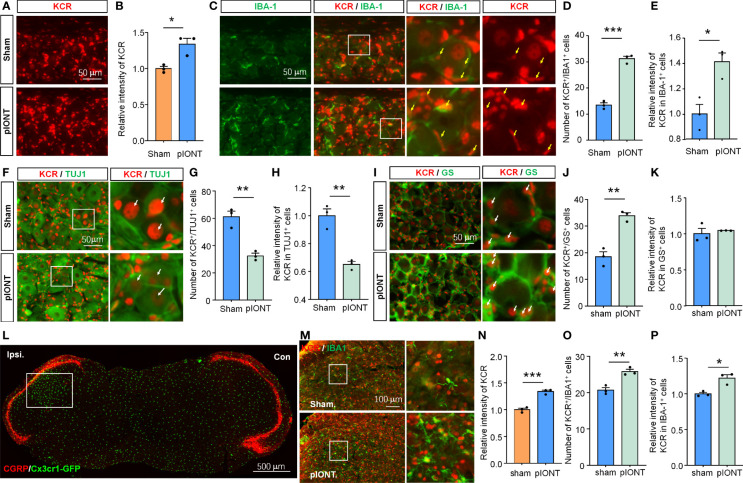
The level of KCR in TG after pIONT was analyzed by quantitative immunofluorescence analysis. **(A, B)** Quantification of the total fluorescence intensity of KCR in sham and pIONT TGs 7 days after surgeries. *P < 0.05, n = 3 mice per group, 3 sections per mice, student’s t-test. **(C)** Representative TG sections double-labeled for IBA-1 and KCR from sham and pIONT groups. Yellow arrowheads point to IBA-1 and KCR double-positive cells, scale bar = 50 μm. **(D, E)** Bar diagram showing the number of KCR and IBA-1 double-positive cells **(D)** and KCR intensity in IBA-1 positive cells **(E)**. *P < 0.05, ***P < 0.001, n = 3 mice per group, 3 sections per mice, student’s t-test. **(F)** Representative fluorescent pictures of TUJ1 and KCR in TG sections. Arrows (white) indicate KCR and TUJ1 double-positive cells, scale bar = 50 μm. **(G, H)** Bar diagram showing the number of KCR and TUJ1 double-positive cells **(G)** and KCR intensity in TUJ1 positive cells **(H)**. **P < 0.01, n = 3 mice per group, 3 sections per mice, student’s t-test. **(I)** Representative immunofluorescent staining of GS and KCR in TG sections. Arrows (white) indicate KCR and GS double-positive cells, scale bar = 50 μm. **(J, K)** Bar diagram showing the number of KCR and GS double-positive cells **(J)** and KCR intensity in GS positive cells **(K)**. **P < 0.01, n = 3 mice per group, 3 sections per mice, student’s t-test. **(L)** CGRP staining of the coronal medulla oblongata section from a *Cx3cr1*-GFP mouse with infraorbital nerve injury indicates peripheral sensory innervation region in the medulla oblongata. The unfilled rectangle represents the activated regions of the medulla oblongata after injury. Scale bar = 500 μm. **(M)** Representative immunofluorescent staining of IBA-1 and KCR of the activated regions of medulla oblongatas from sham and pIONT mice. Box highlights magnified regions. Scale bar = 100μm. **(N–P)** The statistical results of the total fluorescence intensity of KCR **(N)**, the number of KCR and IBA-1 double-positive cells **(O)**, and the intensity of KCR in IBA-1 positive cells. *P < 0.05, **P < 0.01, ***P < 0.001, compared with the sham group. N = 3 mice per group, 3 sections per mice, student’s t-test.

Nerve injury can cause cell morphology and gene expression changes in TG or DRG, which are the biological basis for pain genesis (3, 19). We, therefore, next asked whether KCR levels in different cell types would also vary with them after pIONT. Peripheral nerve injury increased the bulk signal intensity of KCR in TG (**Figures 2A, B**, *P < 0.05, student’s t-test). The number of KCR-positive macrophages and the intensity of KCR in macrophages both increased as macrophages proliferated in TG after pIONT (**Figures 2C–E**, *P < 0.05, ***P < 0.001, student’s t-test), suggesting KCR may be required for macrophage activation and proliferation. We further quantified the fluorescence intensities of KCR in neurons and the number of KCR-positive neurons per unit area of TG sections. However, unlike macrophages, the number of KCR-positive neurons and KCR signals in neurons from the pIONT group were significantly decreased compared to the sham group, suggesting KCR in neurons may be affected by peripheral nerve injury (**Figures 2F–H**, **P < 0.01, student’s t-test). The number of KCR-positive satellite glia was also significantly increased by pIONT (**Figures 2I–K**, **P < 0.01, student’s t-test).

pIONT-induced peripheral nerve injury resulted in medulla oblongata microglial activation in the region innervated by the trigeminal ganglion (**Figure 2L**). The bulk fluorescence intensity of KCR in the activated region of the medulla oblongata after pIONT was also significantly increased compared to that in the Sham group (**Figures 2M, N**, ***P < 0.001, student’s t-test). Additionally, we found that both the number of KCR-positive microglia and KCR signals in microglia from the pIONT group were significantly increased compared to the sham group (**Figures 2M, O, P**; *P < 0.05, **P < 0.01, student’s t-test). Based on the above results, we predicted that KCR might regulate cellular functions of TG macrophage, sensory neurons, and medulla oblongata microglia in response to nerve injury and further modulate pain.

### Inhibition of KCR Attenuates the Mechanical Allodynia Induced by pIONT or Crotonyl-CoA Lithium

**Figure 3:**
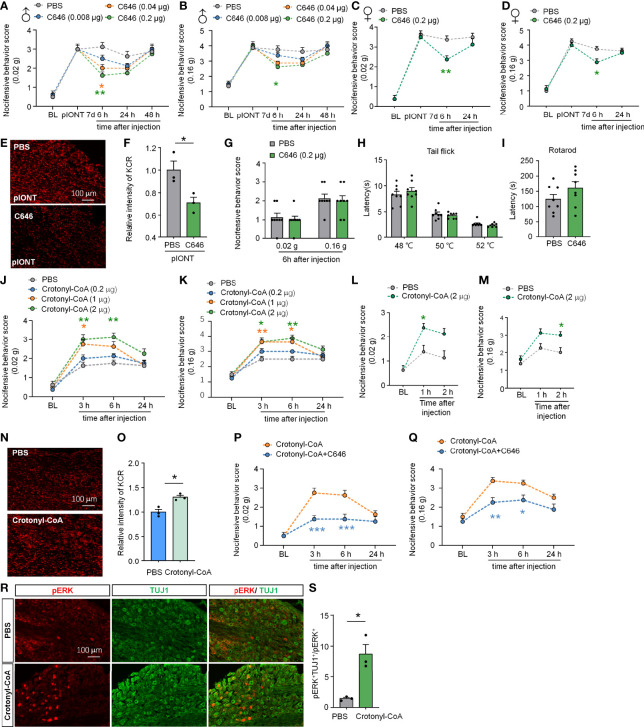
Altering KCR levels in TG affect trigeminal neuropathic pain. **(A, B)** Behavioral results of facial pain in mice under different intensities of mechanical stimulation (0.02 g and 0.16 g). A single dose of intra-TG injection C646 (0.04, 0.2 μg) on day 7 after pIONT significantly attenuated pIONT-induced mechanical allodynia in male mice. The different-colored lines indicate different groups and asterisks of the same color indicated their respective comparison with the PBS group, n = 8 mice per group, *P < 0.05, ** *P* < 0.01, Two-way RM ANOVA followed by Bonferroni’s test. **(C, D)** C646 (0.2 μg) attenuated pIONT-induced mechanical allodynia in female mice 6 hours after pIONT. N = 8 mice per group, *P < 0.05, ** *P* < 0.01, Two-way RM ANOVA followed by Bonferroni’s test. **(E, F)** Representative images and statistics of fluorescence intensity of KCR 6 hours after intra-TG injection of PBS or C646 on pIONT mice. *P < 0.05, N = 3 mice per group, 3 sections per mice, student’s t-test. **(G–I)** Mechanical hyperalgesia **(G)**, thermal hyperalgesia **(H)**, and motor coordination **(I)** were evaluated by von Fery, tail-flick test, and rotarod analysis on naive mice 6 hours after a single intra-TG injection with PBS or C646. N = 8 mice per group. **(J, K)** Crotonyl-CoA Lithium significantly induced facial hyperalgesia in mice in a dose-dependent manner, *P < 0.05, ** *P* < 0.01, n = 8 mice in each group, Two-way RM ANOVA followed by Bonferroni’s test. **(L, M)** Crotonyl-CoA Lithium significantly induced facial hyperalgesia in mice 1-2 hours postinjection. *P < 0.05, n = 8 mice in each group, Two-way RM ANOVA followed by Bonferroni’s test. **(N, O)** Representative images and statistics of fluorescence intensity of KCR 6 hours after intra-TG injection of PBS or Crotonyl-CoA Lithium on naive mice. *P < 0.05, N = 3 mice per group, 3 sections per mice, student’s t-test. **(P, Q)** C646 significantly attenuated Crotonyl-CoA-induced mechanical allodynia when C646 and Crotonyl-CoA Lithium were injected simultaneously, *P < 0.05, **P < 0.01, ***P < 0.001 compared with the Crotonyl-CoA Lithium group, n = 8 in each group, Two-way RM ANOVA followed by Bonferroni’s test. **(R, S)** Representative images and statistics of the number of pERK-positive cells in TG 6 hours after intra-TG injection of PBS or Crotonyl-CoA Lithium on naive mice. *P < 0.05, N = 3 mice per group, 3 sections per mice, student’s t-test.

Given the widespread occurrence of KCR in the TG and medulla oblongata, we wondered whether histone crotonylation is involved in the regulation of neuropathic pain. First, we examined the effect of inhibiting histone crotonylation on pIONT-induced significant facial mechanical allodynia in male mice. Different doses of C646 (0.08, 0.04, 0.2 µg) were administered into TG 7 days after the pIONT. Under mechanical stimulation of 0.02 g, as shown in **Figure 3A**, compared to PBS injection, C646 at 0.2 µg and 0.04 µg significantly attenuated pIONT-induced mechanical nociceptive behavior score by about 36% and 48% in male mice at 6 h after injection (Treatment: F_(3, 28)_ = 6.186, *P* = 0.0023, two-way RM ANOVA followed by the Bonferroni’s test). Similarly, under 0.16 g of mechanical stimulation (**Figure 3B**), 0.2 µg C646 significantly relieved the pain behaviors by 30% in male mice at 6 h (Treatment: F_(3, 28)_ = 4.913, *P* = 0.0072, two-way RM ANOVA followed by the Bonferroni’s test). Next, we further evaluated the effect of C646 (0.2 µg) on pIONT-induced pain in female mice. Under mechanical stimulation of 0.02 g, as shown in **Figure 3C**, C646 at 0.2 µg significantly attenuated pIONT-induced mechanical allodynia in female mice at 6 h after injection (Treatment: F_(1, 14)_ = 8.542, *P* = 0.0111, two-way RM ANOVA followed by the Bonferroni’s test). Under 0.16 g of mechanical stimulation (**Figure 3D**), 0.2 µg C646 significantly relieved the pain behaviors in female mice at 6 h (Treatment: F_(1, 14)_ = 7.177, *P* = 0.0180, two-way RM ANOVA followed by the Bonferroni’s test). Immunofluorescent staining revealed that C646 significantly reduced KCR levels in pIONT TG (**Figures 3E, F**; *P < 0.05, student’s t-test). Finally, we also evaluated whether intra-TG administration of C646 affected naive mice’s mechanical hyperalgesia, thermal hyperalgesia, and motor coordination and found no significant differences (**Figures 3G–I**). These results showed that C646 could alleviate pain behaviors in a dose-dependent manner.

Since Crotonyl-CoA is a substrate for histone crotonylation, administration of Crotonyl-CoA Lithium can increase the KCR level by selectively increasing crotonyl-CoA’s cellular concentrations (9). To investigate whether upregulating KCR causes mechanical hyperalgesia, we injected Crotonyl-CoA Lithium into the TG of naive mice at a series of doses (0.2, 1, 2 µg) and observed their facial pain behavior. The behavioral results in **Figures 3J, K** showed that administration of Crotonyl-CoA Lithium in TG at 2 µg or 1µg could significantly induce mechanical hyperalgesia in mice from 3h to 6h after injection (**Figure 3J**: F_(3, 28)_ = 14.92, *P* < 0.0001; **Figure 3K**: Treatment: F_(3, 28)_ = 13.07, *P* < 0.0001; Two-way RM ANOVA followed by the Bonferroni’s test). In addition, we examined whether Crotonyl-CoA Lithium facilitated pain at shorter time points after injection. Behavioral results showed that Crotonyl-CoA Lithium can induce pain within 2 hours of injection (**Figure 3L**: F_(1, 14)_ = 10.30, *P* = 0.0063; **Figure 3M**: Treatment: F_(1, 14)_ = 13.05, *P* = 0.0028; Two-way RM ANOVA followed by the Bonferroni’s test). Immunofluorescent staining revealed that Crotonyl-CoA Lithium significantly increased the levels of KCR in TG (**Figures 3N, O**; *P < 0.05, student’s t-test). Additionally, we evaluated the inhibitory effect of C646 on Crotonyl-CoA Lithium-induced mechanical hyperalgesia. Using the facial pain behavioral test, we observed that C646 significantly attenuated Crotonyl-CoA Lithium-induced mechanical allodynia from 3 h to 6 h after injection (**Figure 3P**: Treatment: F_(1, 14)_ = 28.00, *P* = 0.0001; **Figure 3Q**: Treatment: F_(1, 14)_ = 18.24, *P* = 0.0008; Two-way RM ANOVA followed by the Bonferroni’s test). We also demonstrated that Crotonyl-CoA Lithium significantly evoked ERK activation in the sensory neurons in TG (**Figures 3R, S**; *P < 0.05, student’s t-test).

Taken together, the results demonstrate that histone crotonylation may be involved in the regulation of nerve injury-induced neuropathic pain. Besides, inhibition of p300 attenuates the mechanical allodynia induced by pIONT or Crotonyl-CoA Lithium, suggesting that p300 may catalyze crotonylation in TG.

### C646 Inhibits pIONT-Induced TG Macrophage Activation and Neuroinflammatory Cytokines and Chemokines Expression

**Figure 4:**
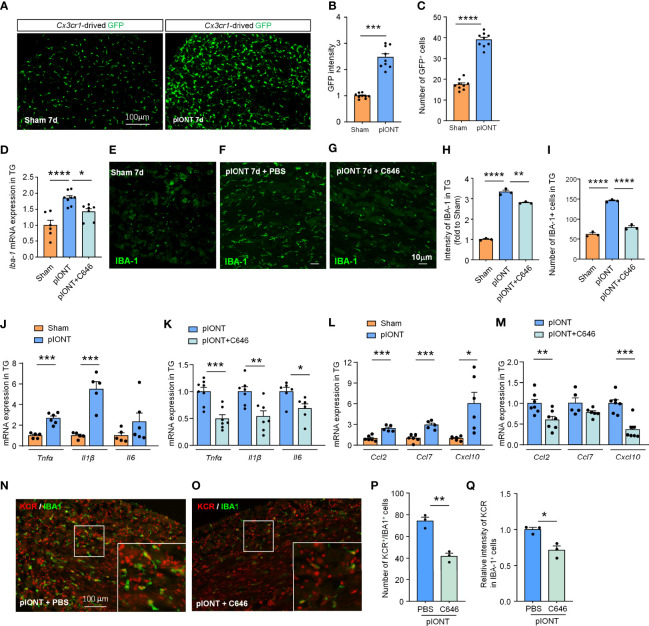
C646 significantly inhibited pIONT-induced macrophage activation and suppressed TG’s upregulation of inflammatory cytokines and chemokines. **(A–C)** *Cx3cr1*-GFP mice were used to visualize the activation of macrophages in TG 7 days after pIONT. The intensity of GFP expression **(B)** and the number of GFP-positive cells **(C)** was significantly increased in the pIONT group. N = 9 sections from 3 mice, each group, *P < 0.05, ****P < 0.0001, one-way ANOVA followed by Bonferroni’s test. **(D)** C646 suppressed the mRNA expression of *Iba-1* induced by pIONT in TG. * *P* < 0.05, ****P < 0.0001, student’s t-test. **(E–I)** Representative confocal images and immunofluorescence analysis data showed IBA-1 protein expression in TG. C646 (1 μg) significantly decreased pIONT-induced IBA-1 expression **(H)** and the number of IBA-1 positive cells **(I)**. N = 3 mice per group, 3 sections per mouse. **P < 0.01, ****P < 0.0001, one-way ANOVA followed by Bonferroni’s test. **(J, K)** The mRNA levels of *Tnfα*, *Il1β* and *Il6* were determined using qRT-PCR. The mRNA expression of *Tnfα* and *Il1β* were significantly increased after pIONT **(J)**, *** *P* < 0.001, compared with the Sham group. However, C646 significantly decreased the mRNA expression of *Tnfα* and *Il1β* upregulated by pIONT **(K)**, *P < 0.05, **P < 0.01, ***P < 0.001, compared with the pIONT group, student’s t-test. **(L, M)** The mRNA levels of *Ccl2*, *Ccl7* and *Cxcl10* were determined using qRT-PCR. C646 significantly decreased the mRNA expression of chemokines upregulated by pIONT. *P < 0.05, **P < 0.01, ***P < 0.001, Student’s t-test. **(N–Q)** Representative images **(N, O)** and statistics of the number of KCR and IBA-1 double-positive cells **(P)** and the fluorescence intensity of KCR in IBA-1 positive cells **(Q)** 6 hours after intra-TG injection of PBS or C646 on pIONT mice. *P < 0.05, **P < 0.01, N = 3 mice per group, 3 sections per mice, student’s t-test.

Nerve injury-induced DRG macrophage activation contributes to pain development or maintenance (26). Our data suggested that TG macrophages are also significantly activated 7 days after pIONT (**Figures 4A–C**, ***P < 0.001, ****P < 0.0001, student’s t-test). Given the increased KCR-positive macrophages in TG after pIONT, we investigated whether the inhibition of histone crotonylation affects the activation of macrophages by monitoring the mRNA level and fluorescence intensity of IBA-1. Here we showed that C646 significantly inhibited pIONT-induced upregulation of IBA-1 mRNA (****P < 0.0001, *P < 0.05, one-way ANOVA followed by Bonferroni’s test, **Figure 4D**) and protein (****P < 0.0001, **P < 0.01, one-way ANOVA followed by Bonferroni’s test, **Figures 4E–H**). The KCR and IBA-1 double-positive cells also significantly decreased by C646 (****P < 0.0001, one-way ANOVA followed by Bonferroni’s test, **Figure 4I**). The above results suggested that KCR might contribute to pIONT-induced TG macrophage expansion. It is known that macrophages in sensory ganglia or microglia in the spinal cord contribute to pain by releasing inflammatory factors and chemokines (19, 27). Hence, we examined the mRNA expression of pain-related proinflammatory cytokines (*Tnfα*, *Il1β* and *Il6*) and chemokines (*Ccl2*, *Ccl7* and *Cxcl10*) by real-time PCR. *Tnfα* and *Il1β* were significantly upregulated 2.67- and 5.49-fold respectively in TG at 10 days after pIONT, compared to that from sham, while *Il6* was unchanged (***P < 0.001, student’s t-test, **Figure 4J**). Expectantly, C646 significantly reversed the expression of *Tnfα* and *Il1β*, and decreased *Il6* (*P < 0.05, **P < 0.01, ***P < 0.001, Student’s t-test, **Figure 4K**). *Ccl2*, *Ccl7* and *Cxcl10* were up-regulated 2.42-, 2.93- and 6.05-fold after pIONT, respectively (*P < 0.05, ***P < 0.001, Student’s t-test, **Figure 4L**). Similarly, the expression of *Ccl2* and *Cxcl10* was significantly reduced by C646 (**P < 0.01, ***P < 0.001, Student’s t-test, **Figure 4M**). Immunofluorescence analyses demonstrated that C646 significantly reduced the KCR and IBA-1 double-positive cells and decreased KCR levels in macrophages (**Figures 4N–Q**, *P < 0.05, **P < 0.01, Student’s t-test). The above results showed that KCR might be necessary for macrophage activation and the expression of inflammatory cytokines and chemokines in TG after peripheral nerve injury.

### C646 Inhibits Crotonyl-CoA-Induced Macrophage Activation and the Neuroinflammatory Cytokines and Chemokines Expression

**Figure 5:**
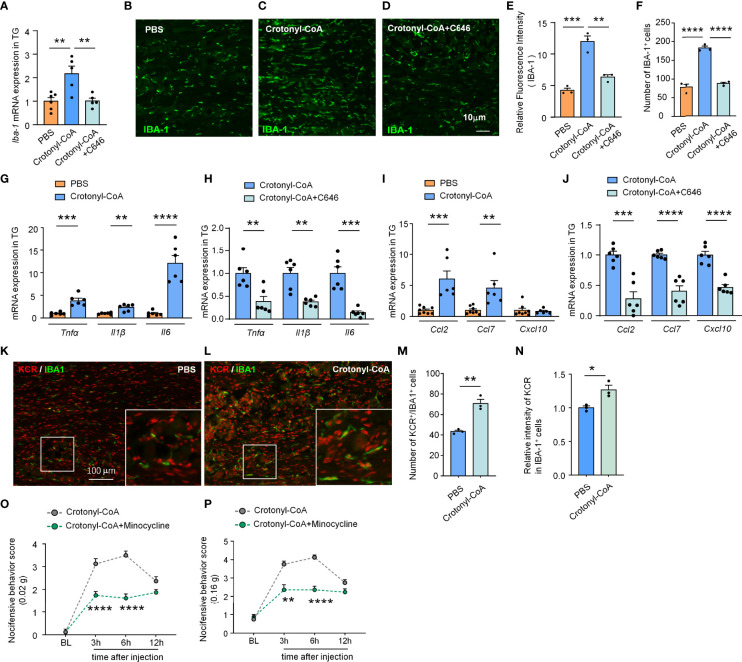
C646 blocked crotonyl-CoA induced macrophage activation and the upregulation of inflammatory factors and chemokine in TG. **(A)** C646 suppressed the upregulation of *Iba1* mRNA induced by Crotonyl-CoA Lithium in TG. ** *P* < 0.01, n = 5-6, One-way ANOVA followed by Bonferroni’s test. **(B–F)** Representative images and Quantitative analysis of immunofluorescence staining showing C646 (1 μg) significantly decreased Crotonyl-CoA Lithium induced IBA-1 upregulation **(E)** and the number of IBA-1 positive cells **(F)** in TG. ** *P* < 0.01, *** *P* < 0.001, ****P < 0.001, One-way ANOVA followed by Bonferroni’s test, n = 3 mice per group, 3 sections per mouse. **(G)** The mRNA levels of *Tnfα*, *Il1β* and *Il6* increased significantly after Crotonyl-CoA Lithium treatment, ** *P* < 0.01, *** *P* < 0.001, ****P < 0.001, n = 6, student’s t-test. **(H)** C646 significantly decreased the mRNA expression of *Tnfα*, *Il1β* and *Il6* upregulated by Crotonyl-CoA Lithium, ** *P *< 0.01, *** *P* < 0.001, compared with the Crotonyl-CoA Lithium group, n = 6, student’s t-test. **(I)** Crotonyl-CoA Lithium significantly increased the mRNA level of *Ccl2* and *Ccl7*, ** *P *< 0.01, *** *P* < 0.001, compared with the PBS group, n = 6-8, student’s t-test. **(J)** C646 significantly decreased the mRNA expression of *Ccl2*, *Ccl7* and *Cxcl10*, *** *P* < 0.001, ****P < 0.0001, compared to Crotonyl-CoA Lithium, n = 6, student’s t-test. **(K–N)** Representative images **(K, L)** and statistics of the number of KCR and IBA-1 double-positive cells **(M)** and the fluorescence intensity of KCR in IBA-1 positive cells **(N)** 6 hours after intra-TG injection of PBS or Crotonyl-CoA Lithium on naïve mice. *P < 0.05, **P < 0.01, N = 3 mice per group, 3 sections per mice, student’s t-test. **(O, P)** Minocycline (1 nmol per mouse) significantly attenuated Crotonyl-CoA-induced mechanical allodynia when minocycline and Crotonyl-CoA Lithium were injected simultaneously, **P < 0.01, ****P < 0.0001 compared with the Crotonyl-CoA Lithium group, n = 8 in each group, Two-way RM ANOVA followed by Bonferroni’s test.

**Figure 6:**
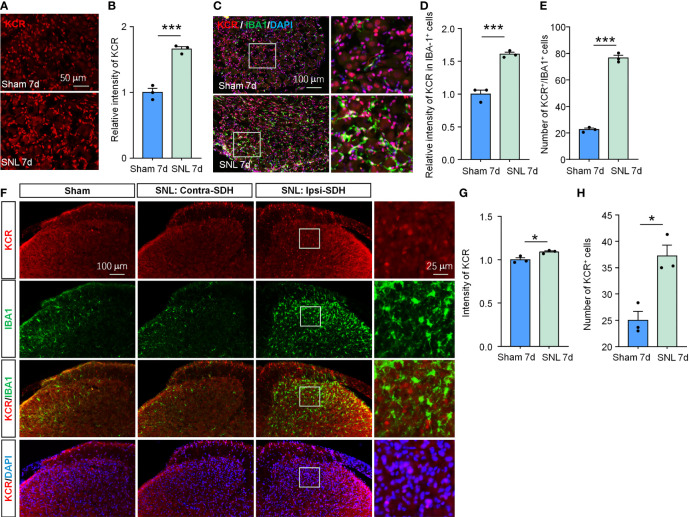
SNL upregulated KCR levels in DRG and spinal cord. **(A, B)** Quantification of the total fluorescence intensity of KCR in sham and SNL DRGs 7 days after surgeries. ***P < 0.001, n = 3 mice per group, 3 sections per mice, student’s t-test. **(C)** Representative DRG sections labeled for DAPI, IBA-1 and KCR from sham and SNL groups. Scale bar = 100 μm. **(D, E)** Bar diagram showing KCR intensity in IBA-1 positive cells **(D)** and the number of KCR and IBA-1 double-positive cells **(E)**. ***P < 0.001, n = 3 mice per group, 3 sections per mice, student’s t-test. **(F)** Representative images labeled for KCR, IBA-1, and DAPI in ipsilateral spinal cords of sham mice or in ipsilateral (SNL: Ipsi-SDH) and contralateral (SNL: Contra-SDH) spinal cords of SNL mice 7 days post-surgeries. The rightmost images are the magnification of the white boxes in the SNL: Ipsi-SDH group. **(G, H)** Quantification of the total fluorescence intensity **(G)** and number **(H)** of KCR in sham- and SNL-spinal dorsal horns seven days after surgeries. *P < 0.05, n = 3 mice per group, 3 sections per mice, student’s t-test.

Next, we further verified if increasing the level of KCR in TG is sufficient to induce macrophage activation and the expression of proinflammatory factors and chemokines. Administration of Crotonyl-CoA Lithium significantly upregulated the mRNA and protein level of IBA-1 in TG of naïve mice, as well as the number of macrophages, and this effect could be reversed by C646 treatment (**P < 0.01, ***P < 0.001, ****P < 0.0001, One-way ANOVA followed by Bonferroni’s test, **Figures 5A–F**), suggesting upregulating KCR level can activate TG macrophage. Similarly, Crotonyl-CoA Lithium upregulated the expression of *Tnfα*, *Il1β* and *Il6* by 3.87-, 2.34- and 12.04 fold, respectively (**P < 0.01, ***P < 0.001, ****P < 0.0001, Student’s t-test, **Figure 5G**). Moreover, these upregulations induced by the Crotonyl-CoA Lithium were significantly reversed by C646 (**P < 0.01, ***P < 0.001, Student’s t-test, **Figure 5H**). *Ccl2* and *Ccl7* were up-regulated 6.03- and 4.59-fold, respectively after Crotonyl-CoA Lithium treatment, while *Cxcl10* was not upregulated (**P < 0.01, ***P < 0.001, Student’s t-test, **Figure 5I**). However, C646 downregulated all the chemokines in TG subjected to Crotonyl-CoA Lithium treatment (***P < 0.001, ****P < 0.0001, Student’s t-test, **Figure 6J**). Immunofluorescence analyses demonstrated that Crotonyl-CoA Lithium significantly increased the KCR and IBA-1 double-positive cells and upregulated KCR levels in macrophages (**Figures 5K–N**, *P < 0.05, **P < 0.01, Student’s t-test). Finally, we validated that macrophage inhibitor minocycline could inhibit Crotonyl-CoA Lithium-evoked pain behavior (**Figure 5O**: F_(1, 14)_ = 42, *P* < 0.0001; **Figure 5P**: Treatment: F_(1, 14)_ = 63.81, *P* < 0.0001; Two-way RM ANOVA followed by the Bonferroni’s test). These results showed that KCR upregulation alone might be sufficient to induce macrophage activation and TG inflammation, which is probably its pain-causing mechanism.

### KCR is Involved in SNL-Induced Neuropathic Pain

Although their respective local peripheral nerve injury ably induced orofacial pain and somatic pain, the cellular composition and gene expression patterns differed between DRG and TG (28). Therefore, the pathogenesis of neuropathic pain occurring on different tissue sites is not identical. Given this, we also evaluated whether KCR is involved in SNL-induced neuropathic pain to confirm further the effects of histone crotonylation in somatic neuropathic pain and the universality of intervention strategies. As in TG, the global KCR level was significantly increased in DRG after SNL (**Figures 6A, B**, ***P < 0.001, student’s t-test). The number of KCR-positive macrophages and the intensity of KCR in macrophages also both increased as macrophages proliferated in DRG after SNL (**Figures 6C–E**, ***P < 0.001, student’s t-test), suggesting KCR may be required for DRG macrophage activation and proliferation during neuropathic pain. In addition, the global KCR level was increased in SDH after SNL (**Figures 6F–H**). Interestingly, the KCR immunofluorescence signal barely co-localized with IBA1 in SDH, which differed from its distribution in TG, DRG and medulla oblongata (**Figure 6F**).

**Figure 7:**
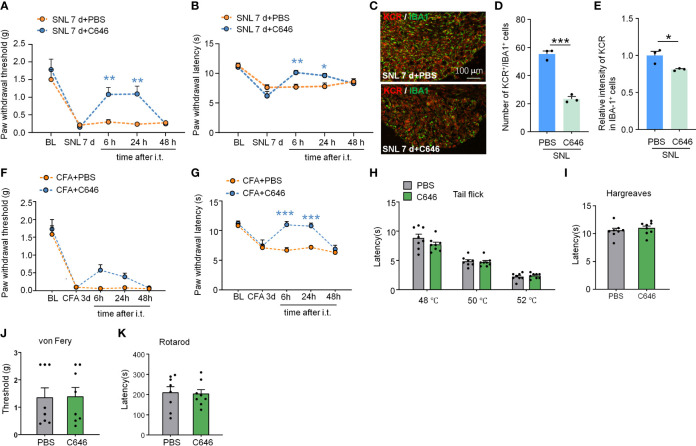
Intrathecal administration of C646 attenuated SNL-induced neuropathic pain and CFA-induced inflammatory pain. **(A, B)** Intrathecal injection of C646 (2 μg) significantly relieved plantar mechanical pain and thermal pain hyperalgesia induced by SNL. *P < 0.05, **P < 0.01, compared with the SNL+PBS group, n = 6-7 mice per group, Two-way RM ANOVA followed by Bonferroni’s test. **(C–E)** Representative images **(C)** and statistics of the number of KCR and IBA-1 double-positive cells **(D)** and the fluorescence intensity of KCR in IBA-1 positive cells **(E)** 6 hours after intra-TG injection of PBS or C646 on SNL mice. *P < 0.05, *** *P* < 0.001, N = 3 mice per group, 3 sections per mice, student’s t-test. **(F, G)** Intrathecal injection of C646 significantly relieved plantar mechanical pain and thermal pain hyperalgesia induced by CFA. ***P < 0.001, compared with the SNL+PBS group, n = 8 mice per group, Two-way RM ANOVA followed by Bonferroni’s test. **(H–K)** Naive mice that received an intrathecal injection of C646 exhibited normal thermal sensitivity **(H, I**, tail-flick test and Hargreaves test**)**, mechanical sensitivity **(J**, *von* Frey test**)**, and motor coordination **(K**, rotarod test**)**.

Intrathecal delivery is a feasible and non-invasive way to affect DRG cells mainly. We thus downregulated the KCR level in DRG by intrathecal injection of C646. Pain behavior results indicate that, on day 7 after SNL, C646 was able to relieve mechanical allodynia and heat hyperalgesia significantly at 6 hours and 24 hours after intrathecal injection (**Figure 7A**: Treatment: F_(1, 11)_ = 6.995, *P* = 0.0228; **Figure 7B**: Treatment: F_(1, 11)_ = 2.364, *P* = 0.1524. Two-way ANOVA followed by the Bonferroni’s test). Immunofluorescence analyses demonstrated that in DRG, C646 significantly reduced the KCR and IBA-1 double-positive cells and decreased KCR levels in macrophages (**Figures 7C–E**, *P < 0.05, ***P < 0.001, Student’s t-test). Robust macrophage/microglial activation occurred in the DRG/spinal cord during the intraplantar CFA-induced inflammatory main (29). Behavioral data showed that C646 also significantly reduces CFA-induced mechanical allodynia and thermal hyperalgesia in mice (**Figure 7F**: Treatment: F_(1, 14)_ = 4.921, *P* = 0.0436; **Figure 7G**: Treatment: F_(1, 14)_ = 19.55, *P* = 0.0006. Two-way ANOVA followed by the Bonferroni’s test). Same as the intra-TG injection, the intrathecal injection of C646 did not affect naive mice’s mechanical sensitivity, thermal sensitivity, and motor coordination (**Figures 7H–K**). These results suggested that KCR in DRG might be involved in the genesis of SNL-induced somatic neuropathic pain and CFA-induced inflammatory pain *via* mediating macrophage activation.

**Figure 8:**
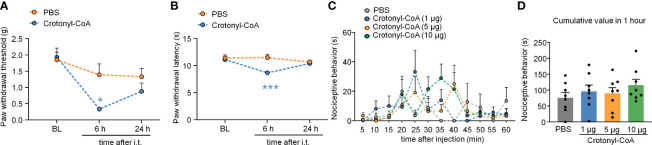
Intrathecal administration of Crotonyl-CoA induced pain sensitivity. **(A, B)** Intrathecal injection of Crotonyl-CoA markedly induced plantar mechanical pain and thermal hyperalgesia in mice. *P < 0.05, *** *P* < 0.001, compared with the PBS group, n = 6-7 mice per group, Two-way RM ANOVA followed by Bonferroni’s test. **(C, D)** Mice that received an intraplantar injection of different doses of Crotonyl-CoA did not exhibit nociceptive behavior. N = 8 mice per group, Two-way RM ANOVA **(C)** or one-way ANOVA **(D)** followed by Bonferroni’s test.

We also upregulated KCR level in DRG by intrathecal injection of Crotonyl-CoA Lithium (2 μg per mouse) in normal animals. Compared with the PBS-injected group, Crotonyl-CoA Lithium induced significantly mechanical allodynia and heat hyperalgesia at 6 h after injection (**Figure 8A**: Treatment: F_(1, 11)_ = 6.093, *P* = 0.0312; **Figure 8B**: Treatment: F_(1, 11)_ = 5.868, *P* = 0.0339. Two-way RM ANOVA followed by the Bonferroni’s test). However, the intraplantar injection of Crotonyl-CoA Lithium in doses of 1, 5, and 10 μg per mouse did not elicit nociceptive behavior (**Figures 8C, D**).

## Discussion

**Figure 9:**
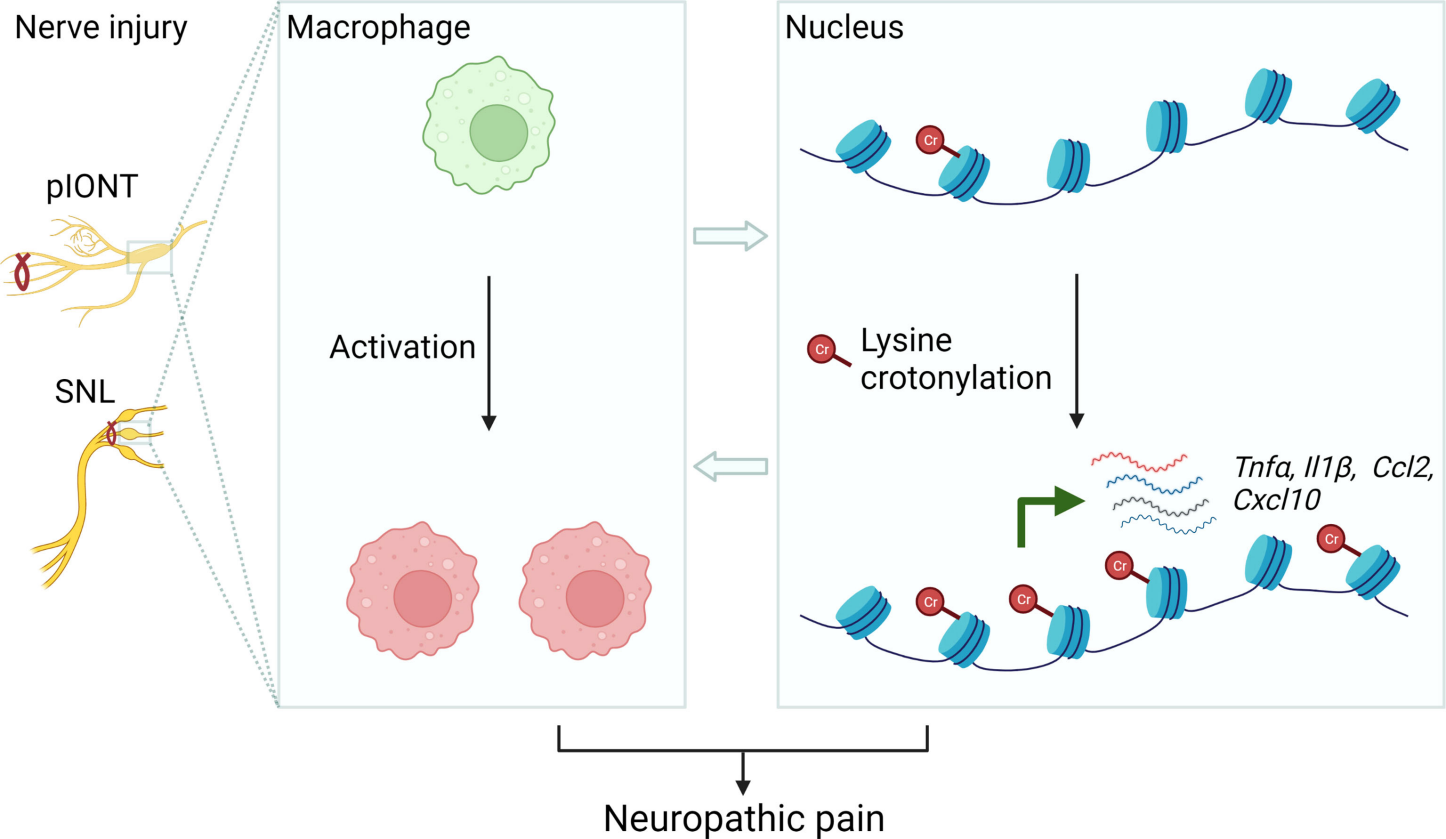
Schematic diagram illustrating the possible mechanism of KCR in peripheral nerve injury-induced neuropathic pain. The left panel represents establishing the facial and somatic neuropathic pain animal models. The middle panel illustrates that peripheral nerve injury induces macrophage activation in TG or DRG. The right panel schematically indicates histone lysine crotonylation increased in macrophages after injury, resulting in the transcriptional enhancement of *Tnfα*, *Il1β*, *Ccl2* and *Cxcl10*. These processes ultimately cause neuropathic pain.

In recent years, the role of histone lysine crotonylation in development and disease is now gaining increasing attention (8). For the first time, our data demonstrated that KCR, a prevalent lysine modification in histone, occurred in neuronal and non-neuronal cells in TG, DRG medulla oblongata and spinal cord. Peripheral nerve injury contributed to KCR level upregulation in macrophages but downregulation in sensory neurons in TG. Decreasing KCR by inhibiting its writer p300 reduced pain and the production of multiple mediators of inflammation. Additionally, exogenously administered crotonyl-CoA induced mechanical hyperalgesia in mice and increased the expression of inflammatory cytokines and chemokines (**Figure 9**). Although preliminary, current results provided intriguing findings that histone lysine crotonylation was involved in the regulation of neuropathic pain and neuroinflammation, providing new prospects for treating neuropathic pain.

With the advancement of high-resolution liquid chromatography with tandem mass spectrometry (LC-MS/MS), recent studies have revealed that besides methylation, ubiquitination, and simulation, the protein lysine residues can be reversibly modified by various acyl groups, including formylation, acetylation, and propionylation butyrylation, succinylation, myristoylation, glutarylation and crotonylation (10). KCR is a conserved and widespread post-translational modification that has been detected from yeast to humans, from germline to somatic cells (7, 30). The present study provides evidence that KCR occurs in satellite glial cells, neurons, and macrophages of DRG or TG; In the medulla oblongata, it occurs in microglial cells, neurons, and astrocytes. Histone lysine crotonylation was mainly distributed in nuclei of mammalian cells and occurred predominantly on histone at the promoter regions near the transcription start sites (TSS) and enhancer regions (7). In agreement with previous studies, our results showed that the KCR immunofluorescence signal is confined to the nucleus, regardless of cell types in sensory ganglia or medulla oblongata. Such features of KCR on peripheral and central systems suggest that it is likely to be involved in the transcriptional regulation in these cells, although no specific sites and mechanisms of KCR have been identified yet.

Although KCR may have complex and pleiotropic effects in different cell types and conditions, we wondered about the overall physiological effect of global upregulated or downregulated KCR levels on pain regulation, which is important given the treatment of pain. Indeed, abundant evidence shows that various types of histone acylation, including lysine acetylation and crotonylation, can occur non-enzymatically when the concentration of cellular acyl groups remains high (13, 31). Several studies have proved that exogenous crotonyl-CoA could upregulate KCR levels of histone both *in vitro* and *in vivo* (9, 13). We tested the role of KCR on pain by intra-TG or intrathecal injection of Crotonyl-CoA Lithium that is able to supply crotonyl-CoA for cells *in vivo* (9, 12). Behavioral data indicated that Crotonyl-CoA Lithium induced mechanical and thermal hyperalgesia sufficiently in facial or somatic neuropathic pain.

Given that KCR mainly exerts promoting transcription effects, we speculate that after peripheral nerve injury, the elevated KCR levels in macrophages may induce pain by upregulating the expression of pain-causing genes, and reduced KCR levels in the sensory neurons may contribute to pain genesis by downregulating the anti-nociceptive genes, such as GABA receptors or potassium channels encoding genes (4, 32). Consistent with this speculation, intra-TG injection of Crotonyl-CoA Lithium upregulated the expression of pain-promoting genes *Tnfa*, *Il1β*, *Il6*, *Ccl2* and *Ccl7*. C646 is a selective inhibitor that selectively blocks p300/CBP family, which has previously been shown to have crotonyltransferase activity (9, 33). Although p300 has both crotonyltransferase and acetyltransferase activities, p300-catalyzed histone crotonylation presents more direct pro-transcriptional effects than histone acetylation (9). We demonstrated that C646 could reduce nerve injury- or crotonyl-CoA-induced neuropathic pain, suggesting that C646 may exert analgesic effects by reducing TG’s KCR level. Given that C646 and most current analgesics target different molecules, we speculate they may produce additive effects when used together in neuropathic pain. However, the deficiency is that the effects of C646 on histone deacetylation cannot be ruled out. Altogether, these data provide the potential to target KCR for the treatment of neuropathic pain.

Nerve injury-induced neuroinflammation in DRG and spinal cord has been demonstrated to play a pivotal role in the pathogenesis of neuropathic pain, notably by the activation of local innate immune cells and the upregulation of cytokines or chemokines (20, 26, 27, 29). Our experimental results showed that exogenous crotonyl-CoA could significantly upregulate the expression of proinflammatory genes like *Tnfa*, *Il1β*, *Il6*, *Ccl2* and *Ccl7* in TG, and their upregulation induced by nerve injury could be suppressed by concomitant administration of C646. Consistent with it, intra-TG administration of crotonyl-CoA mimicked peripheral nerve injury-induced TG macrophage activation, and C646 could inhibit these activations. Another significant point that should be noted here is that the KCR level was upregulated in TG macrophages after pIONT. Arguably, our current results revealed that upregulation of KCR contributed to macrophage activation and exacerbated neuroinflammatory response. Therefore, we speculate that nerve injury may influence the modification levels of KCR that may contribute to pain *via* upregulating inflammation-related genes. Further research should be undertaken to elucidate the detailed molecular mechanisms KCR regulates inflammatory gene expression. Next, we discuss the inter-regulatory relationship between KCR and inflammation. Our results and previous evidence indicate that enhanced KCR levels are closely associated with inflammation and tissue injury (33). Lipopolysaccharides (LPS) stimulation upregulated the KCR level at the promoters of inflammatory genes and promoted their expression in macrophages (9). Besides, these inflammatory gene expressions were enhanced by p300 *via* further potentiating KCR (9). KCR level, in turn, is also regulated by inflammation. One study showed that kidney KCR was globally increased in the mouse model of the folic acid- or cisplatin-induced acute kidney injury (AKI). The proinflammatory cytokine TWEAK, a driver of kidney injury, also increased KCR level in cultured proximal tubule epithelial cells (15). Therefore, a bidirectional positive feedback regulation may be between KCR and inflammation.

In short, our study provided the first evidence that KCR is widely distributed in different cell types in the sensory ganglia, medulla oblongata and SDH. We also demonstrated that histone crotonylation regulates neuropathic pain and neuroinflammation. Therefore, targeting enzymes that modify KCR to promote its crotonylation or decrotonylation is likely a viable therapeutic strategy for neuropathic pain or diseases associated with neuroinflammation.

## Data Availability Statement

The raw data supporting the conclusions of this article will be made available by the authors, without undue reservation.

## Ethics Statement

The animal study was reviewed and approved by the Ethics Committee of Nantong University.

## Author Contributions

YZ, TD, and LK performed quantitative PCR, behavioral tests, and immunostaining. XB, PZ, F-FX and FD participated in the immunostaining and behavioral experiments. YL analyzed the data. BJ designed the experiments, analyzed the data, coordinated and supervised the project, and wrote the manuscript.

## Funding

This study was supported by grants from the National Natural Science Foundation of China (81971054, 81771197, 81874171) and the Jiangsu Qinglan Project.

## Conflict of Interest

The authors declare that the research was conducted in the absence of any commercial or financial relationships that could be construed as a potential conflict of interest.

## Publisher’s Note

All claims expressed in this article are solely those of the authors and do not necessarily represent those of their affiliated organizations, or those of the publisher, the editors and the reviewers. Any product that may be evaluated in this article, or claim that may be made by its manufacturer, is not guaranteed or endorsed by the publisher.

## Abbreviations

pIONT, partial infraorbital nerve transection; SNL, spinal nerve ligation; TG, trigeminal ganglia; DRG, dorsal root ganglia; KCR, histone lysine crotonylation; IBA-1, Ionized calcium-binding adapter molecule 1; GS, glutamine synthetase; TUJ1, neuron-specific class III beta-tubulin; DAPI, 4’,6-diamidino-2-phenylindole; NF200, neurofilament protein 200; CGRP, calcitonin gene-related peptide; IB4, Isolectin B4; GFAP, glial fibrillary acidic protein; NeuN, neuronal nuclei; pERK, phosphorylated extracellular signal-regulated kinase; *Tnfα*, tumor necrosis factor α; *Il1β*, interleukin 1β; *Il6*, interleukin 6; *Ccl2*, C-C motif chemokine ligand 2; *Ccl7*, C-C motif chemokine ligand 7; *Cxcl10*, C-X-C motif chemokine ligand 10; PWL, paw withdrawal latency.
